# Editorial: Introducing Registered Reports

**DOI:** 10.1523/ENEURO.0089-18.2018

**Published:** 2018-03-14

**Authors:** Christophe Bernard

The way we do science is rather stereotyped. We have hypotheses to test, and we plan the experiments, sometimes a long time ahead, to test these hypotheses. Writing a grant proposal is a typical example: we give a detailed plan of what we are going to do in the coming years. Of course, we never know how a project will evolve, and deviations from the original plan are commonplace, as milestones may not be reached. But in many instances, the hypothesis is such that deviations rarely occur. Preclinical studies are a prototypical example: we test whether a drug can treat a particular disorder. The experimental plan is straightforward. Unavoidably, many projects lead to dead ends, negative results, etc.

Publishing such negative results is important for the advancement of science, as it saves time and money for labs tempted to test the same hypotheses, and allows researchers to move on to testing other hypotheses. This is the reason why the Negative Results category of papers exists at *eNeuro*.

There is another way to deal with this issue: Registered Reports, a research publishing format started by the Center for Open Science. The principle is simple. You can submit a text that contains a short introduction to provide the background and why testing this hypothesis is important, as well as a detailed Materials and Methods section, to show how you are going to test the hypothesis. This text is then reviewed to evaluate the validity of the hypothesis and whether the experimental plan addresses the raised questions. If approved, the resulting paper (after the experiment is completed) can be accepted whatever the results will be, i.e., positive or negative.

This pre-evaluation has two main advantages:

1. It provides useful feedback to authors before they start performing the experiments. In that way, the project can be greatly improved. This ultimately saves time and money. I would personally enjoy feedback from peers at very early stages of a project, as one cannot always see potential caveats, or better ways to demonstrate what we want to show. External advice is always important.
2. It guarantees publication, again saving time, as the study is already provisionally accepted.

I am pleased to announce *eNeuro* will launch Registered Reports on March 14, 2018. How will it work? As mentioned above, submit the Introduction and the detailed Materials and Methods section. This is reviewed by a reviewing editor and two reviewers and follows a procedure similar to a regular paper, i.e., reviewers may accept it as is, request a revision, or reject it.

If the Registered Report is accepted, then the authors complete the study and submit the full paper containing the usual sections. The paper goes through expedited reviewing and will be accepted whatever the results are, provided that there is no deviation from the original plan. Deviations from the provisionally approved methodology are acceptable, but then the paper will go through a regular evaluation process.

It is important to note that having the paper accepted as a Registered Report at *eNeuro* does not bind the authors to *eNeuro*. After finishing the experimental work, authors are free to submit the paper anywhere. However, if a Registered Report is withdrawn after provisional acceptance, *eNeuro* will publish a notice of this withdrawal, to indicate that the study had been evaluated by us, this is only fair.

From personal experience, I know that it is sometimes impossible to predict how a project will exactly evolve in time. This is the nature of science. However, in some instances, the experimental plan can be straightforward, like for preclinical studies. Therefore, we will first limit Registered Reports to preclinical studies designed to test the effect of a treatment. However, if you think that your nonclinical experimental plan will not deviate over time, you can contact me about it, I would be happy to consider any type of study in the Registered Report category.

*eNeuro*: always striving to help the community

